# Adoption of routine telemedicine in Norway: the current picture

**DOI:** 10.3402/gha.v7.22801

**Published:** 2014-01-09

**Authors:** Paolo Zanaboni, Undine Knarvik, Richard Wootton

**Affiliations:** 1 Norwegian Centre for Integrated Care and Telemedicine, University Hospital of North Norway, Tromsø, Norway; 2 Faculty of Health Sciences, University of Tromsø, Tromsø, Norway

**Keywords:** telemedicine, adoption, routine use, implementation, decision making

## Abstract

**Background:**

Telemedicine appears to be ready for wider adoption. Although existing research evidence is useful, the adoption of routine telemedicine in healthcare systems has been slow.

**Objective:**

We conducted a study to explore the current use of routine telemedicine in Norway, at national, regional, and local levels, to provide objective and up-to-date information and to estimate the potential for wider adoption of telemedicine.

**Design:**

A top-down approach was used to collect official data on the national use of telemedicine from the Norwegian Patient Register. A bottom-up approach was used to collect complementary information on the routine use of telemedicine through a survey conducted at the five largest publicly funded hospitals.

**Results:**

Results show that routine telemedicine has been adopted in all health regions in Norway and in 68% of hospitals. Despite being widely adopted, the current level of use of telemedicine is low compared to the number of face-to-face visits. Examples of routine telemedicine can be found in several clinical specialties. Most services connect different hospitals in secondary care, and they are mostly delivered as teleconsultations via videoconference.

**Conclusions:**

Routine telemedicine in Norway has been widely adopted, probably for geographical reasons, as in other settings. However, the level of use of telemedicine in Norway is rather low, and it has significant potential for further development as an alternative to face-to-face outpatient visits. This study is a first attempt to map routine telemedicine at regional, institutional, and clinical levels, and it provides useful information to understand the adoption of telemedicine in routine healthcare and to measure change in future updates.

Telemedicine, the use of communications networks for delivering healthcare services and medical education from one geographical location to another (1), has been shown to work, to be effective in many clinical areas (2, 3), to be sustainable (4), and, in some cases, to be cost-effective (5). As a consequence, telemedicine appears to be ready for wider adoption in healthcare systems (6). Decisions about the institutionalization of telemedicine are usually taken by healthcare regulators, governments, and authorities (7), and research findings are useful to support evidence-based policy making (8). Despite the great potential of telemedicine, its adoption in routine healthcare has been slow, uneven, and fragmented (9, 10). Several telemedicine applications appear to be promising candidates for widespread use, but they remain at the early adoption stage (11). Implementation has often been problematic (12), and utilization remains limited in many settings (13), with a consequent disconnection between policy, practice, and research (14–16).

Norway is a country with a long interest in telemedicine, where testing of new applications has been conducted since the 1990s in the form of pilot projects or small-scale services (17). A survey conducted in 1998 identified 102 telemedicine programs in Norway, mainly in the radiology, psychology, dermatology, pathology, and otolaryngology specialties. Some of the programs had passed through the project phase and become fully operational, others had lacked staff with the motivation and time to carry them further, and finally some were deemed to be of limited use (18). During the 2000s, significant resources were directed to the development of telemedicine services, with the aim of achieving high-quality and cost-effective healthcare. In 2003, the Norwegian Centre for Telemedicine (NST) conducted a survey of telemedicine activities in Norway. The survey showed variations between health regions with respect to both the type and the number of activities, and it found that telemedicine was provided as a routine service only to a minor degree (19). In 2005, the Northern Norway Regional Health Authority initiated a review to clarify which telemedicine services could be escalated into large-scale use. Candidates for large-scale implementation included teleradiology, digital communication and integration of patient records and education, teledialysis, emergency medicine, telepsychiatry, teledermatology, pediatrics, district medical centers, teleophthalmology, and teleotorhinolaryngology (20). More recently, the Norwegian Ministry of Health commissioned the NST to recommend telemedicine services which were ready for large-scale implementation in the health service, together with the necessary actions to secure a successful dissemination of the services (21). Despite this monitoring activity, a comprehensive picture of the current adoption and level of use of routine telemedicine in Norway is still missing. Moreover, a framework for reporting telemedicine utilization at national and global levels is still lacking.

We conducted a study to explore the current adoption and utilization of routine telemedicine in the Norwegian healthcare system at national, regional, and local levels, and to discuss the potential for wider adoption. Routine use is defined as an application which has entered everyday use, typically after a minimum of 1 year in service (22). We did not focus on telemedicine services implemented as pilot projects. The findings of this study aim to bridge the gap between research, policy, and practice (23), thus supporting both decision makers about defining proper action strategies to integrate telemedicine in healthcare systems and practitioners about implementing it in routine clinical practice.

## Methods

We used two different and complementary approaches to collect information about routine telemedicine in Norway. A top-down approach was used to collect official data on the national use of telemedicine from the Norwegian Patient Register (NPR). A bottom-up approach was used to collect complementary information through a survey conducted at the largest publicly funded hospitals in Norway.

### National data

The NPR is a central health registry that was established in 1997 and is run by the Norwegian Ministry of Health. The NPR was established to provide data for planning, evaluation, and financing of publicly funded specialized healthcare, and also for medical and health services research. The NPR contains data regarding outpatient care delivered by hospitals with activity-based financing. Therefore, only telemedicine contacts that are reimbursed to hospitals are included. In Norway, a ‘telemedicine contact’ is defined as the use of videoconferencing to perform an outpatient consultation, examination, or treatment at a distance. This means that the contact between patient and physician takes place simultaneously in two different locations (24). The use of store-and-forward telemedicine, such as the transmission of still images, is not covered by any reimbursement scheme in Norway. Contacts occurring by telephone, short message service (SMS), or similar means are not considered as telemedicine contacts. The reimbursement of a telemedicine contact delivered via videoconference is equal to that of a traditional outpatient visit.

A formal request was sent to the NPR in February 2012 to obtain data regarding telemedicine activities. This study did not involve human participants. We collected anonymized information on the number of telemedicine contacts. No personally identifiable data related to individuals were collected. Ethical approval from the Regional Ethics Committees and consent were therefore not required, according to the Norwegian Health Research Act (25) and the Personal Data Act (26). The request was approved by the Norwegian Ministry of Health, and completed data were delivered in August 2012. Data were stratified for health regions (northern Norway, central Norway, western Norway, and south-eastern Norway), by hospital, by year (2009, 2010, and 2011), and by clinical specialty. Adoption was expressed as the percentage of the number of adopters over the potential users (27). In addition, the potential number of cases where telemedicine could be used instead of a traditional face-to-face visit was estimated. Data on the number of outpatient visits were thus collected from the NPR.

### Local data

Since data from the NPR only cover telemedicine contacts for which a reimbursement is possible (i.e. where videoconferencing is used), we conducted a survey at the largest publicly funded hospitals in Norway to collect complementary information on the routine use of telemedicine. We selected five hospitals in the four health regions: the University Hospital North Norway in northern Norway, the St. Olavs University Hospital in central Norway, the Haukeland University Hospital and the Stavanger University Hospital in western Norway, and the Oslo University Hospital in south-eastern Norway. All of these hospitals are hospital trusts and are connected with local hospitals.

In our survey, telemedicine services were classified according to the type of technology or modality into:

1. Synchronous services (use of videoconferencing);
2. Asynchronous services (use of email and/or other electronic messaging); or
3. Both synchronous and asynchronous services;

and according to the nature of the consultation into:

1. A telemedicine consultation (assessment, evaluation, treatment, or management of a patient) between a specialist and another specialist, a doctor, and/or other health personnel; or
2. A home-monitoring service between the patient and doctor.

First contact was made at the Regional Health Authority, the information and communications technology (ICT) department, the clinical divisions, or the integrated care department. A list of people involved in the provision of telemedicine (clinicians, nurses, advisors, ICT professionals, and heads of departments) was then provided by our first contact. At the University Hospital of North Norway, due to the presence of the NST as a competence center, the survey was based on a list of telemedicine services and contact persons provided internally. The data collection period lasted from May 2012 to the end of November 2012.

The contacts identified were asked to answer a questionnaire, either through interview or by email, which was structured as follows:

1. Medical field
2. Short description of the telemedicine service
3. Is it a routine service or a project?
4. Which other institutions are involved?
5. How long has the service existed (approximately)?
6. How many times did you provide the telemedicine service in 2011 (approximately)?
7. When was the last time you provided a telemedicine service?
8. Is it a synchronous or asynchronous service, or both?
9. Is it a telemedicine consultation or a home-monitoring service?

The survey did not involve collecting data about human participants. We collected anonymized and qualitative information on routine services. No personally identifiable data related to individuals were collected, and no information was obtained from the Electronic Health Record (EHR) systems of the hospitals. According to the Norwegian Health Research Act (25) and the Personal Data Act (26), ethical approval and consent were thus not required for the survey. All interviewees were asked and approved that the results of the survey would be publicly available and published.

## Results

### National data

Data regarding the routine use of telemedicine contacts were available for 2009, 2010, and 2011. The overall use of telemedicine followed an irregular trend over 3 years. The level of use decreased considerably from 2009 to 2010, and then it increased again in 2011 (Table 1). All four health regions in Norway reported use of telemedicine, with a consequent 100% adoption at the regional level. In 2011, 54% and 45% of the overall telemedicine contacts were reported in northern Norway and western Norway, respectively, thus representing almost the whole national activity. Central Norway and south-eastern Norway showed low levels of activity. In 2011, there were 4.9 million outpatient visits in the four health regions. There were 1,827 telemedicine episodes (i.e. 0.04% of the total). Northern Norway reported the highest relative use of telemedicine contacts, according to the total number of face-to-face visits.

**Table 1:** Outpatient visits and telemedicine contacts in the period 2009–2011 in the four health regions

Health region	Outpatient visits (2009)	Outpatient visits (2010)	Outpatient visits (2011)	Telemedicine contacts (2009)	Telemedicine contacts (2010)	Telemedicine contacts (2011)
Western Norway	879,911	930,840	947,303	240 (0.03%)	246 (0.03%)	821 (0.09%)
Central Norway	695,161	724,617	763,467	449 (0.06%)	23 (0.00%)	1 (0.00%)
Northern Norway	470,078	484,151	502,839	1,739 (0.37%)	876 (0.18%)	986 (0.20%)
South-Eastern Norway	2,573,532	2,625,076	2,711,593	318 (0.01%)	41 (0.00%)	19 (0.00%)
Total	4,618,682	4,764,684	4,925,202	2,746 (0.06%)	1,186 (0.02%)	1,827 (0.04%)

The four health regions deliver healthcare services through 28 hospitals (Appendix 1). Nineteen out of 28 hospitals reported that they had used telemedicine during the 3-year period, with a consequent 68% adoption at the institutional level. However, some hospitals did not use telemedicine continuously over the period. The number of hospitals providing more than 50 telemedicine contacts every year decreased from nine in 2009 to only three in 2010 and 2011. The University Hospital of North Norway in northern Norway and the Stavanger University Hospital in western Norway covered most of the telemedicine contacts in 2011 (90%). These two hospitals had also the highest relative use of telemedicine compared to the total number of face-to-face outpatient visits. The other hospitals delivering routine telemedicine provided little telemedicine activity.

The main use of telemedicine was for neurosurgery (Table 2). Neurosurgery was the only clinical area with a relatively high use of telemedicine compared to the total number of outpatient visits (5.5% in 2009). Other clinical specialties also reported the use of telemedicine, but only in the range of a few hundred cases every year. These included physical medicine and rehabilitation, cardiovascular diseases, eye diseases, skin and venereal diseases, female diseases and obstetrics, children's diseases, and orthopedics. A lower level of telemedicine activity was reported for the remaining clinical areas. The use of telemedicine in relation to the clinical specialties differed from hospital to hospital (Appendix 2). Six hospitals had 10 or more clinical areas involved in the provision of routine telemedicine.

**Table 2:** Outpatient visits and telemedicine contacts in the period 2009–2011 in different clinical specialties

Clinical specialties	Outpatient visits (2009)	Outpatient visits (2010)	Outpatient visits (2011)	Telemedicine contacts (2009)	Telemedicine contacts (2010)	Telemedicine contacts (2011)
Neurosurgery	14,701	16,858	19,144	805 (5.48%)	385 (2.28%)	469 (2.45%)
Physical medicine and rehabilitation	190,645	201,525	222,851	388 (0.20%)	162 (0.08%)	789 (0.35%)
Clinical neurophysiology	32,330	34,874	51,313	353 (1.09%)	3 (0.01%)	7 (0.01%)
Skin and venereal diseases	197,707	204,552	189,415	221 (0.11%)	239 (0.12%)	93 (0.05%)
Eye diseases	228,680	266,363	287,130	154 (0.07%)	137 (0.05%)	231 (0.08%)
Cardiovascular diseases	222,183	228,739	249,516	152 (0.07%)	58 (0.03%)	51 (0.02%)
Children's diseases	205,979	208,943	191,248	119 (0.06%)	25 (0.01%)	5 (0.00%)
Obstetrics	482,661	502,400	513,143	102 (0.02%)	18 (0.00%)	14 (0.00%)
Oncology and radiotherapy	94,258	196,281	206,750	71 (0.08%)	9 (0.00%)	2 (0.00%)
Orthopaedics and rheumatology	647,839	708,595	739,050	60 (0.01%)	72 (0.01%)	76 (0.01%)
Urology	135,833	148,557	160,630	49 (0.04%)	9 (0.01%)	17 (0.01%)
General surgery	166,569	146,575	133,809	35 (0.02%)	10 (0.01%)	7 (0.01%)
General internal medicine	68,642	65,136	61,923	27 (0.04%)	1 (0.00%)	1 (0.00%)
Digestive diseases	122,480	142,525	162,385	23 (0.02%)	1 (0.00%)	11 (0.01%)
Endocrinology	108,866	117,577	119,423	16 (0.01%)	2 (0.00%)	5 (0.00%)
Pulmonary diseases	100,842	112,729	118,963	16 (0.02%)	4 (0.00%)	20 (0.02%)
Neurology	111,310	115,714	133,537	16 (0.01%)	9 (0.01%)	13 (0.01%)
Ear, nose, and throat diseases	324,964	333,776	353,326	15 (0.00%)	9 (0.00%)	3 (0.00%)
Hematology	61,811	75,488	84,666	14 (0.02%)	0 (0.00%)	0 (0.00%)
Kidney diseases	53,312	52,288	54,164	12 (0.02%)	3 (0.01%)	3 (0.01%)
Maxillofacial and mouth diseases	29,634	27,554	26,746	10 (0.03%)	2 (0.01%)	0 (0.00%)
Geriatrics	16,338	16,931	18,427	10 (0.06%)	0 (0.00%)	0 (0.00%)
Gastroenterological surgery	114,129	123,111	126,741	9 (0.01%)	4 (0.00%)	1 (0.00%)
Other clinical specialties	944,532	778,658	736,326	69 (0.01%)	24 (0.00%)	9 (0.00%)
Total	4,676,245	4,825,749	4,960,626	2,746 (0.06%)	1,186 (0.02%)	1,827 (0.04%)

### Local data

A total of 75 telemedicine services were identified in the survey. Of these, 50 were excluded because they were not routine services but still pilot projects. Of the remaining 25 routine telemedicine services, eight were operational at the University Hospital of North Norway, three at the St. Olavs University Hospital, eight at the Haukeland University Hospital, two at the Stavanger University Hospital, and four at the Oslo University Hospital. The 25 routine services were implemented in several clinical fields (Table 3). Most routine telemedicine was delivered through synchronous services via videoconference. The remaining services were asynchronous or used a combination of both synchronous and asynchronous technologies.

**Table 3:** Routine telemedicine services in different clinical fields

Clinical fields	Routine telemedicine services	Synchronous services	Asynchronous services	Synchronous and asynchronous services
Dermatology	3	1	1	1
Emergency medicine	3	1		2
Pathology	3	2	1	
Pulmonary medicine	3	2		1
Neurology (stroke)	1	1		
Cardiology	2		2	
Psychiatry	2	2		
Oncology	2	2		
Orthopaedics	2	2		
Nephrology	1	1		
Gynecology and obstetrics	1			1
Maritime medicine	1			1
Surgery	1			1
Total	25	14	4	7

These telemedicine services were provided from the large hospitals to institutions at different healthcare levels (Table 4). Most services (12 out of 25) operated with other hospitals at the secondary care level, either belonging to the same health trust or in other health trusts. The second category of telemedicine services (8 out of 25) operated at the primary care level, serving general practitioners, district medicine centers, nursing homes, and homecare. The remaining services were provided to oil platforms and ships.

**Table 4:** Routine telemedicine services in different types of institutions

Type of institutions involved	Number of routine services
Hospitals in the same health trust	2
Hospitals in other health trusts	8
Hospitals in the same health trust and hospitals in other health trusts	2
General practitioners	3
District medical centers, nursing homes, and hospitals in the same or other health trusts	3
District medical centers and nursing homes	2
Home-monitoring services with patients	2
Oil platforms	2
Ships	1
Total	25

Some routine telemedicine services had been in use for more than 15 years (Table 5). Maritime medicine has been used in Norway since 1949, when medical guidance was given by telephone, and since the early 1990s images have been sent from ships to hospitals for diagnosis. Teledermatology started in 1998, when dermatological images were first sent by general practitioners to hospitals. Telepathology has existed since the late 1990s. Few telemedicine services (four out of 25) became operational in 2012.

**Table 5:** Routine telemedicine services and year of establishment

Period of establishment (year established)	Number of routine services
<1 year (2012)	4
>1 to 4 years (2008–2012)	9
<5 to 7 years (2005–2007)	6
<8 to 10 years (2002–2004)	2
>10 years (before 2001)	4
Total	25

The majority of routine telemedicine services (21 out of 25) provided teleconsultations for the assessment, evaluation, treatment, or management of patients. Three services were delivered as home monitoring, while one service consisted of both teleconsultations and home monitoring.

#### Telemedicine services in secondary care

In pulmonary medicine, videoconferencing was used, together with clinical data on lung function and CT images, for the assessment of patients with lung diseases in local hospitals. Specialists analyzed this information and made suggestions about additional examinations, treatment, and time for surgery. A similar service was used in cardiology, in which electrocardiography (ECG) and coronary angiography were evaluated. In oncology, decision support regarding chemotherapy treatment and symptom management was provided to local hospitals and district medicine centers via videoconference. Videoconferencing was also used by radiotherapy departments to communicate with other hospitals for assessment, procedures, and administration of patient cases. The same principle for decision support and second opinions was provided by hospitals within several fields, such as gynecology and obstetrics, where the patient pathway (e.g. pre-operative assessment) and distribution of tasks were discussed, and patient's information (e.g. X-rays) exchanged electronically. In pathology, videoconferencing was used to discuss kidney biopsy cases between hospitals. Pathology departments could also send sections from digital microscopes for postoperative diagnosis of cancer. In neurology, telestroke was used to assess patients’ conditions after acute stroke and support thrombolysis. In nephrology, renal departments at specialist hospitals provided teledialysis to remote satellite dialysis units without nephrologists via videoconference. In dermatology, for instance, second opinions were provided by a specialist hospital to a rehabilitation hospital via videoconference for the assessment of patients with bedsores. In psychiatry, regional hospitals used videoconferencing to cooperate regarding the admission of patients to the regional security department. A decentralized on-call service via videoconference was used for adults with mental health problems, and a similar service was provided to regional child and adolescent psychiatry centers. Finally, the surgical department of one hospital offered a service for burn injuries with national responsibility in this field to other national hospitals. Digital images of the damaged area were transmitted electronically, and specialists gave advice on whether patients should be transferred and on which type of treatment should be provided. The numbers of teleconsultations reported are summarized in Table 6.

**Table 6:** Routine telemedicine services at the five largest hospitals in Norway

Hospital	Clinical field	Type of service	Teleconsultations (2011)	Institutions involved	Year of establishment
University Hospital of North Norway	Cardiology	Asynchronous	250	Home monitoring	2008
	Oncology	Synchronous	90	Hospitals in other health trusts	2007
	Oncology	Synchronous	60	General practitioners and hospitals in other health trusts	2009
	Psychiatry	Synchronous	40–50	Hospitals in the same health trust	2009
	Emergency medicine	Both	5–10	District medicine and hospitals in the same health trust	2005
	Dermatology	Both	30–100	General practitioners, home nursing, and hospitals	1998
	Nephrology	Synchronous	98	Hospitals in the same and other health trusts	2002
	Orthopaedics	Synchronous	90	District medicine	2007
St. Olavs University Hospital	Emergency medicine	Synchronous	n.a.	Oil platforms	2012
	Pathology	Asynchronous	50	Hospitals in other health trusts	2012
	Orthopaedics	Synchronous	240	District medicine	2005
Haukeland University Hospital	Cardiology	Asynchronous	350	Hospitals in the same and other health trusts	2008
	Gynecology	Both	40–50	Hospitals in other health trusts	2002
	Psychiatry	Synchronous	10	Hospitals in other health trusts	2010
	Maritime medicine	Both	500	Ships	Early 1990s
	Surgery	Both	80	Hospitals in other health trusts	1998
	Emergency medicine	Both	15–20	Oil platforms	2009
	Neurology	Synchronous	20	Hospitals in the same health trust	2009
	Pathology	Synchronous	10	Hospitals in other health trusts	1997
Stavanger University Hospital	Pulmonary medicine	Synchronous	730	Home monitoring	2010
	Dermatology	Asynchronous	50	General practitioners	2012
Oslo University Hospital	Pulmonary medicine	Synchronous	500	Hospitals in other health trusts	2007
	Pulmonary medicine	Both	100	Hospitals in other health trusts	2007
	Pathology	Synchronous	2	Hospitals in other health trusts	2011
	Dermatology	Synchronous	4	Hospitals in other health trusts	2012

#### Telemedicine services for primary care and homecare

In pulmonary medicine, a decentralized telemedicine service was provided for chronic obstructive pulmonary disorder (COPD) patients at risk of exacerbation. Patients were monitored remotely with a briefcase equipped with a laptop, alarms, a spirometer, and a pulse oximeter, and via two-way videoconference. In 2011, 730 teleconsultations were performed by one hospital for 73 patients. In cardiology, telemedicine services were provided for patients with disturbance in heart rhythm and severe heart failure, and for patients with implantable defibrillators and pacemakers, serving approximately 300 patients per year. In orthopedics, videoconferencing was provided to evaluate patients before or after a hospital stay near their homes. Another service was offered to a district medical center for patients injured or undergoing surgery. X-rays were transferred electronically and integrated into the Electronic Patient Record (EPR), and teleconsultations with specialists allowed referrals and follow-up via videoconference. Teledermatology is one of the oldest telemedicine services in Norway, and it is used by general practitioners to send digital images to specialists for diagnosis and treatment. Another service was provided by an outpatient clinic for wound treatment in home nursing. Pictures of leg ulcers taken with digital cameras or mobile phones were sent together with assessments and questions through an Internet-based program.

#### Telemedicine services for oil platforms and ships

Telemedicine was used in emergency medicine by hospitals to support 34 oil platforms in the North Sea with diagnostic help, clinical evaluation, and guidance. Communication was provided via videoconference, and biological parameters were transmitted and visible on monitors at both sites. Telemedicine was also used to provide medical support to ships over large distances, serving approximately 1,000 patients per year. Previous transfer of images has been replaced by videoconferencing, which is used by the doctor on call.

## Discussion

### Potential for wider adoption and utilization

While all the four health regions in Norway reported the use of routine telemedicine, its adoption at the institutional level reaches 68%. Due to the long experience in implementing telemedicine services nationally and due to current evidence of telemedicine's benefits globally, we believe that telemedicine in Norway can be potentially adopted by all 28 publicly funded hospitals, which represent all the potential users in secondary care. Most telemedicine contacts (90%) included in the registry were provided by two hospitals in northern Norway and western Norway, as confirmed by the survey. This might be the result of contextual factors, such as long distances, which characterize those regions. We therefore assume that the level of use of telemedicine is generally higher in countries or areas characterized by major geographical needs. Despite telemedicine being widely adopted in Norway, its level of use locally is still low. Data at the institutional level (Appendix 1) show that the delivery of telemedicine contacts represents up to about 1% of the total number of face-to-face outpatient visits. In most hospitals, however, the relative level of use of telemedicine is much lower. A few examples about the use of telemedicine teleconsultations compared to face-to-face visits can be used as reference. In neurology, store-and-forward telemedicine via email was used to handle just over one-half (54%) of the GP referrals (28). In dermatology, for trials using videoconferencing, the mean proportion of patients who avoided travel was approximately 70%, while for trials using store-and-forward telemedicine it was 43% (29). These data suggest that there is the potential to avoid a substantial proportion – maybe even half – of face-to-face visits to outpatient clinics by using telemedicine. Several of the Norwegian hospitals delivering routine telemedicine have services in 10 or more clinical areas. This means that when telemedicine is adopted by a hospital, it is more likely that services are offered through the whole organization. Neurosurgery is the field with the highest relative use of telemedicine, where the delivery of telemedicine contacts represents about 5% of the total number of face-to-face outpatient visits. This level might, in principle, represent a potential target by hospitals in other clinical areas.

**Fig. 1:**
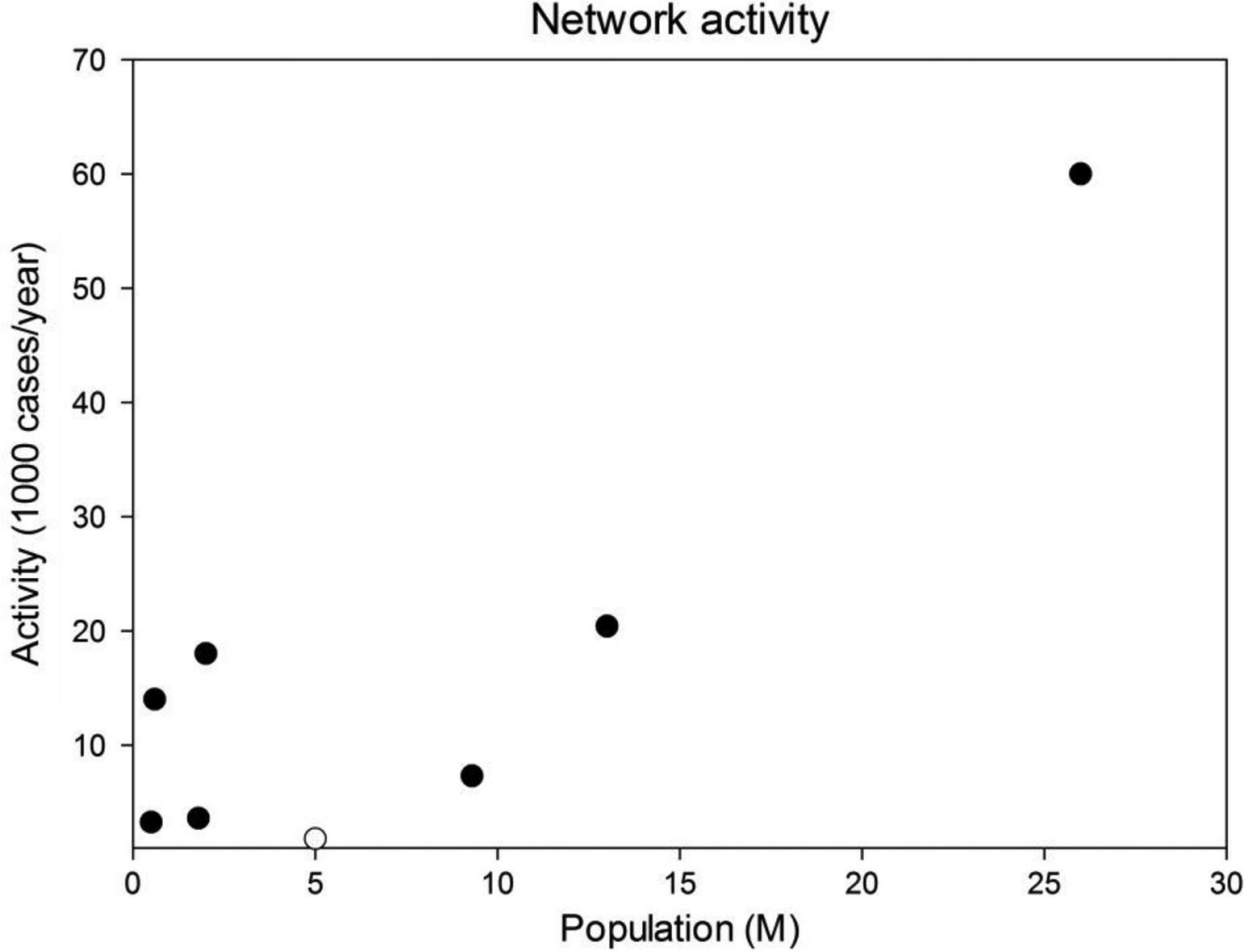
Comparison of telemedicine activity among different statewide networks. Closed symbols are non-Norwegian networks; the open symbol is Norway.

There are few studies regarding the routine adoption of telemedicine in healthcare systems around the world, and no comprehensive data on telemedicine activities in Norway have been published before. In a recent study, Mars and Scott (30) identified 210 reports of telemedicine services, 77 of which provided sufficient data to calculate the number of consultations per site per week as a metric to compare the level of usage. The average use was low: 1.8 consultations per site per week. Data collected from the NPR show an average use of routine telemedicine in Norway of 2.7 consultations per healthcare organization per week in 2011, but the rate per site was not available. We also compared (Table 7) the use of routine telemedicine in Norway with that in other statewide telemedicine networks (31–37). The use of telemedicine in Norway is low compared to that in other, similar contexts (Fig. 1). Assuming the same rate of teleconsultations per inhabitant as in the Ontario Telemedicine Network (34) or the Alaska Federal Health Care Access Network (32), the use of routine telemedicine in Norway could increase to 78,500 or 116,665 teleconsultations every year, respectively. International comparisons regarding telemedicine activity can also be useful in terms of clinical specialties. In a recent systematic survey of the state of teledermatology in the United States, the median number of teleconsultations per site in 2011 was 3,650 and 1,200 for health maintenance organizations (HMOs) and government-associated programs (38), respectively, a higher use compared to routine teledermatology in Norwegian hospitals (Table 6).

**Table 7:** Use of telemedicine in different statewide networks

Paper	Region	Index year	Population served (millions)	Teleconsultations (per year)
Alkmim et al., 2012	Minas Gerais, Brazil	2011	9.3	7,300
Blanchet, 2005	Texas, USA	2005	26	60,000
Brewer et al., 2011	Georgia, USA	2009	2	18,000
Brown, 2013	Ontario, Canada	2011	13	204,000
Kokesh et al., 2011	Alaska, USA	2009	0.6	14,000
Meyers et al., 2012	Nebraska, USA	2010	1.8	3,600
	Norway	2011	5	1,800

### Routine telemedicine services

The NPR has the principal advantage of being a centralized database at the national level, thus making possible systematic, reliable, and efficient data collection from all publicly funded hospitals. However, data from the registry only cover telemedicine contacts for which a reimbursement is possible (i.e. where videoconferencing is used). Our survey provided complementary, more qualitative, and detailed information on the routine use of telemedicine. The numbers of teleconsultations identified in the survey are consistent with those collected from the NPR. The University Hospital of North Norway, for instance, reported 848 episodes in the NPR (Table 2) and about 700 in the survey (Table 6) in 2011. This means that the results from the survey are representative of the current use of telemedicine at the five hospitals.

Most routine telemedicine services identified in the five largest public hospitals connected hospitals at the same healthcare level, while there were fewer telemedicine services connecting secondary care with primary care (e.g. general practitioners) and only limited routine telemedicine in home monitoring. Despite the lack of comparable data globally, we believe that the preponderance of specialist telemedicine services is common in many countries. Moreover, the majority of the telemedicine services were synchronous and used videoconferencing, while fewer services were asynchronous. This might stem from the reimbursement scheme currently in use in Norway, which represents an implementation factor. However, asynchronous services in telemedicine technologies are also used to support decentralized services, offering to specialists more flexibility related to response time as well as a more optimized basis for decision making.

One of the telemedicine services with the highest activity was provided for pulmonary medicine by the largest Norwegian hospital (Oslo University Hospital) (Table 6). This service consisted of teleconsultations via videoconferencing with six minor hospitals in the same region to evaluate patients for lung surgery. Two home-monitoring services can also be distinguished due to high volumes. The Haukeland University Hospital in the Western Norway Regional Health Authority remotely followed 370 patients with implantable cardioverter defibrillators (ICDs) at home. ICDs are monitored automatically, and specialists can access patient data from any available PC. Another home-monitoring service was used by the Stavanger University Hospital in the Western Norway Regional Health Authority to follow up on COPD patients discharged from the hospital, to prevent readmissions, and to detect exacerbations early. A well-used service (both synchronous and asynchronous) was run by the Norwegian Centre for Maritime Medicine at the Haukeland University Hospital. Radio Medico Norway has been providing medical guidance to ships by telephone since 1949, currently serving 1,000–1,200 patients a year. In the 1990s, most commercial ships were equipped with a digital camera, and transfer of pictures via e-mail became an efficient support mechanism for medical diagnoses. In August 2012, videoconferencing was installed at the center and on a mobile PC to be used by the doctor on call, while ships were provided with ultrasound, ECG, an ophthalmoscope, and a high-definition camera.

### Implementation factors

Although analysis of the factors affecting adoption is beyond the scope of the present study, we speculate that the routine use of telemedicine can be related to implementation factors, such as organizational, contextual, and policy issues, which act as facilitators, or barriers, for the integration of telemedicine in healthcare organizations and clinical practices (13, 39–41). Over the years, Norway has developed several plans for the promotion of ICT in healthcare. These strategies supported investment in standardization (e.g. EPRs), with a consequent positive impact on the further implementation of telemedicine. Videoconferencing between healthcare institutions is delivered inside the Norwegian Health Network, which provides a high level of security. The routine use of videoconferencing by the health personnel can be observed by the growing number of these services. The diffusion of picture-archiving and communications systems (PACS) in hospitals made teleradiology an integral part of routine clinical practice, so that today the term ‘teleradiology’ is not in use any longer. Reimbursement policies represent another facilitator, or barrier, for the diffusion of telemedicine services. Norway was the first country to develop a specific reimbursement for telemedicine in 1996, for both synchronous and asynchronous services, with a consequent rise of telemedicine activities in the subsequent decade. However, more recent policies limit reimbursement to the use of videoconferencing, thus affecting the trend regarding use of telemedicine activities in the last few years. In addition, the current reimbursement scheme does not produce any incentive for general practitioners, which represents a further obstacle to the implementation of telemedicine, especially for services connecting secondary care with primary care. These considerations are in line with findings from other studies. The lack of success of telepsychiatry in Australia, for instance, might be due to both insufficient reimbursement and the consequence of not addressing other conditions required for the successful integration of telemedicine into routine clinical practice (42). In contrast, the provision of teledermatology in the United States is supported by different payment methods, including private payers, self-payers, Medicaid, Medicare, HMOs, and contract-based services (38).

### Limitations

We acknowledge some limitations in our study. Data in the NPR contain only telemedicine contacts for which a reimbursement was possible (i.e. where videoconferencing was used) and a request for payment was forwarded to the regional authority. All other telemedicine activities (e.g. store-and-forward) are excluded. Moreover, comparable data were available for only 3 years. As a consequence, we were not able to identify a longer term trend of telemedicine activities over the last decade. The main limitation regarding the survey was related to the volume of routine telemedicine services identified. We decided to conduct the survey at the five largest hospitals from the four health regions in Norway. However, there are also other smaller hospitals which deliver routine telemedicine. These can be included in future work. Moreover, hospitals in Norway do not currently have a formal overview of their telemedicine services, thus making a survey difficult, especially for large and complex healthcare organizations such as those included in this study. However, after conducting this study, the hospitals became aware of the importance of having a clear picture of their telemedicine activity and are willing to have this included in future updates.

### Future implications and framework for reporting of telemedicine utilization

The present study is a first attempt to map national routine telemedicine with objective and up-to-date information of its usage at regional, institutional and clinical levels. Previously, such a comprehensive picture in Norway was missing, and a framework for reporting of telemedicine utilization at the national and global levels was lacking. Moreover, little published data exist for international comparison, and no other study has reported comparable details. The dearth of similar data from other countries made it difficult to report our results for international comparison. It would therefore be premature to comment on the rate of telemedicine usage in Norway relative to that in other countries or networks (Fig. 1). Further work is needed to obtain more comprehensive data from around the world, from both high-income countries and low- and middle-income countries, and to monitor the uptake of telemedicine from year to year in those countries.

We believe that future studies would be much facilitated by recording and reporting data in a standardized fashion. This study provides a baseline to measure changes in future updates to give a more complete picture on the adoption of telemedicine in the Norwegian healthcare system and to report further implementation of telemedicine services. The methodology used in this study can be extended to other countries, including low- and middle-income countries, to provide additional insights about how the adoption of routine telemedicine can vary across different healthcare systems and policies, thus contributing to a common platform which can inform and support evidence-based health policy and practice, both locally and globally.

Good-quality and timely data from health information systems are the foundation of all health systems. However, too often data sit in reports, on shelves, or in databases and are not sufficiently utilized in policy and program development, improvement, strategic planning, and advocacy (43). Where available, central health registries can provide useful and detailed data regarding the use of routine telemedicine nationally. We therefore recommend the use of these sources. However, the example of the NPR shows that these data might cover only part of the overall telemedicine usage. A framework for reporting telemedicine utilization, including a combination of different methods (e.g. quantitative and qualitative), allows one not only to assess more aspects but also to corroborate the findings, which results in more robust conclusions (44). Where limited data are available from health information systems, a survey conducted at the hospital level is able to embrace the whole spectrum of telemedicine activities which are part of the routine practice of healthcare professionals. A survey based on standardized questionnaires can be used to identify routine telemedicine services and to collect, for each service, a standard minimum dataset including the medical field, a short description of the telemedicine service, the type of technology or modality (synchronous, asynchronous, or both), the type of equipment and technical requirements, the nature of the consultation (e.g. telemedicine consultation or a home-monitoring service), the level of implementation (e.g. a routine service or project), the institutions involved, the first year of operation, the average caseload (telemedicine episodes) in the previous year, and the last provision of the telemedicine service. Due to country-dependent issues, information regarding organizational, contextual, and policy issues must also be reported together with data from registries and surveys to facilitate international comparison and detect similarities and dissimilarities between high-income countries and low- and middle-income countries, which may have different resources and healthcare systems.

## Conclusions

The present study provides objective and up-to-date information regarding the adoption of routine telemedicine in Norway at regional, institutional, and clinical levels. We reported both quantitative data collected through a national registry, and more qualitative and detailed data regarding routine telemedicine services collected through a survey. Routine telemedicine in Norway has been widely adopted. The percentage of hospitals adopting telemedicine is high, and examples of routine telemedicine can be found in several clinical specialties. However, the level of use of telemedicine in Norway is rather low, with significant potential for further development as an alternative to face-to-face outpatient visits.

Our research findings contribute to bridging the gap between policy and practice regarding telemedicine services and therefore improving the current debate concerning the routine use of telemedicine in healthcare systems. On one hand, decision makers, governments, and healthcare authorities can monitor and take effective policy actions about the adoption and utilization of telemedicine services at the healthcare system level. On the other hand, health practitioners can be informed better about the benefits of adopting telemedicine in their practice and access targeted incentives which can result in an increase in the number of outpatient visits performed through telemedicine. We believe that these actions might lead to a positive change in the future use of routine telemedicine in Norway, thus moving from face-to-face visits to teleconsultations via videoconferencing as has occurred in some settings elsewhere.

## Appendix

**Appendix 1:** Outpatient visits and telemedicine contacts in the period 2009–2011 in the hospitals

Hospitals	Outpatient visits (2009)	Outpatient visits (2010)	Outpatient visits (2011)	Telemedicine contacts (2009)	Telemedicine contacts (2010)	Telemedicine contacts (2011)
Western Norway						
Stiftelsen Betanien Bergen	1,675	2,059	2,097	0 (0.00%)	0 (0.00%)	0 (0.00%)
Haugesund Sanitetsforenings Hospital for Rheumatic Diseases	21,914	21,066	18,082	2 (0.01%)	0 (0.00%)	0 (0.00%)
Stavanger University Hospital	236,601	274,315	268,052	124 (0.05%)	201 (0.07%)	806 (0.30%)
Fonna Hospital Trust	115,059	118,160	117,049	103 (0.09%)	41 (0.03%)	13 (0.01%)
Bergen Hospital Trust	376,996	388,058	409,798	0 (0.00%)	1 (0.00%)	2 (0.00%)
Førde Hospital Trust	110,630	109,995	112,956	11 (0.01%)	2 (0.00%)	0 (0.00%)
Haraldsplass Diakonale Hospital Trust	17,036	17,187	19,269	0 (0.00%)	1 (0.01%)	0 (0.00%)
Central Norway						
St. Olavs Hospital Trust	327,390	350,338	368,701	448 (0.14%)	23 (0.01%)	1 (0.00%)
Nord Trøndelag Hospital Trust	100,797	99,562	109,382	0 (0.00%)	0 (0.00%)	0 (0.00%)
Møre og Romsdal Hospital Trust	266,974	274,717	285,384	1 (0.00%)	0 (0.00%)	0 (0.00%)
Northern Norway						
Finnmark Hospital Trust	55,048	54,132	55,108	14 (0.03%)	33 (0.06%)	39 (0.07%)
University Hospital of North Norway	214,538	227,831	235,486	1,325 (0.62%)	780 (0.34%)	848 (0.36%)
Nordland Hospital Trust	122,723	126,532	130,953	147 (0.12%)	63 (0.05%)	99 (0.08%)
Helgeland Hospital Trust	77,769	75,656	81,292	253 (0.33%)	0 (0.00%)	0 (0.00%)
South-Eastern Norway						
Betanien Hospital	14,868	16,983	18,760	0 (0.00%)	0 (0.00%)	0 (0.00%)
Sunnaas Hospital Trust	2,691	3,922	3,598	0 (0.00%)	4 (0.10%)	5 (0.14%)
Vestre Viken Hospital Trust	287,427	277,960	296,535	0 (0.00%)	3 (0.00%)	1 (0.00%)
Lovisenberg Diakonale Hospital	43,071	45,088	52,065	10 (0.02%)	0 (0.00%)	0 (0.00%)
Martina Hansens Hospital	22,934	22,964	25,021	0 (0.00%)	0 (0.00%)	0 (0.00%)
Lillehammer Hospital for Rheumatic Diseases	10,701	10,803	12,351	0 (0.00%)	0 (0.00%)	0 (0.00%)
Diakonhjemmet Hospital	51,898	55,768	62,841	0 (0.00%)	0 (0.00%)	0 (0.00%)
Akershus University Hospital	175,830	185,536	233,530	0 (0.00%)	0 (0.00%)	0 (0.00%)
Innlandet Hospital Trust	317,634	320,325	327,537	97 (0.03%)	14 (0.00%)	1 (0.00%)
Østfold Hospital Trust	200,674	195,314	196,563	137 (0.07%)	5 (0.00%)	2 (0.00%)
Sørlandet Hospital Trust	267,781	271,263	279,041	74 (0.03%)	15 (0.01%)	8 (0.00%)
Vestfold Hospital Trust	196,826	195,674	205,989	0 (0.00%)	0 (0.00%)	2 (0.00%)
Telemark Hospital Trust	155,306	164,000	169,598	0 (0.00%)	0 (0.00%)	0 (0.00%)
Oslo University Hospital	825,891	859,476	828,164	0 (0.00%)	0 (0.00%)	0 (0.00%)
Total	4,618,682	4,764,684	4,925,202	2,746	1,186	1,827

**Appendix 2:** Numbers of clinical specialties delivering telemedicine in the hospitals

Hospitals	Clinical specialties (2009)	Clinical specialties (2010)	Clinical specialties (2011)
Western Norway	27	19	12
Stiftelsen Betanien Bergen	0	0	0
Haugesund Sanitetsforenings Hospital for Rheumatic Diseases	1	0	0
Stavanger University Hospital	9	7	2
Fonna Hospital Trust	15	9	8
Bergen Hospital Trust	0	1	2
Førde Hospital Trust	2	1	0
Haraldsplass Diakonale Hospital Trust	0	1	0
Central Norway	7	2	1
St. Olavs Hospital Trust	6	2	1
Nord Trøndelag Hospital Trust	0	0	0
Møre og Romsdal Hospital Trust	1	0	0
Northern Norway	48	33	34
Finnmark Hospital Trust	8	11	10
University Hospital of North Norway	19	8	9
Nordland Hospital Trust	16	14	15
Helgeland Hospital Trust	5	0	0
South-Eastern Norway	21	20	10
Betanien Hospital	2	0	0
Sunnaas Hospital Trust	0	1	1
Vestre Viken Hospital Trust	0	3	1
Lovisenberg Diakonale Hospital	0	0	0
Martina Hansens Hospital	0	0	0
Lillehammer Hospital for Rheumatic Diseases	0	0	0
Diakonhjemmet Hospital	0	0	0
Akershus University Hospital	0	0	0
Innlandet Hospital Trust	9	6	1
Østfold Hospital Trust	4	4	2
Sørlandet Hospital Trust	6	6	3
Vestfold Hospital Trust	0	0	2
Telemark Hospital Trust	0	0	0
Oslo University Hospital	0	0	0
Total	103	74	57

## References

[1] Sood S, Mbarika V, Jugoo S, Dookhy R, Doarn CR, Prakash N (2007). What is telemedicine? A collection of 104 peer-reviewed perspectives and theoretical underpinnings. Telemed J E Health.

[2] Ekeland AG, Bowes A, Flottorp S (2010). Effectiveness of telemedicine: a systematic review of reviews. Int J Med Inform.

[3] Wootton R (2012). Twenty years of telemedicine in chronic disease management – an evidence synthesis. J Telemed Telecare.

[4] Whitten P, Holtz B, Nguyen L (2010). Keys to a successful and sustainable telemedicine program. Int J Technol Assess Health Care.

[5] Whited JD (2010). Economic analysis of telemedicine and the teledermatology paradigm. Telemed J E Health.

[6] Doarn CR, Merrell RC (2008). A roadmap for telemedicine: barriers yet to overcome. Telemed J E Health.

[7] Zanaboni P, Lettieri E (2011). Institutionalizing telemedicine applications: the challenge of legitimizing decision-making. J Med Internet Res.

[8] Hebert MA, Korabek B, Scott RE (2006). Moving research into practice: a decision framework for integrating home telehealth into chronic illness care. Int J Med Inform.

[9] Hanney SR, González-Block MA (2009). Evidence-informed health policy: are we beginning to get there at last?. Health Res Policy Syst.

[10] Wade V, Eliott J, Karnon J, Elshaug AG (2010). A qualitative study of sustainability and vulnerability in Australian telehealth services. Stud Health Technol Inform.

[11] Zanaboni P, Wootton R (2012). Adoption of telemedicine: from pilot stage to routine delivery. BMC Med Inform Decis Mak.

[12] Murray E, Burns J, May C, Finch T, O'Donnell C, Wallace P (2011). Why is it difficult to implement e-health initiatives? A qualitative study. Implement Sci.

[13] Gagnon MP, Duplantie J, Fortin JP, Landry R (2006). Implementing telehealth to support medical practice in rural/remote regions: what are the conditions for success?. Implement Sci.

[14] Jansen MW, van Oers HA, Kok G, de Vries NK (2010). Public health: disconnections between policy, practice and research. Health Res Policy Syst.

[15] Miller EA (2007). Solving the disjuncture between research and practice: telehealth trends in the 21st century. Health Policy.

[16] Cusack CM, Pan E, Hook JM, Vincent A, Kaelber DC, Middleton B (2008). The value proposition in the widespread use of telehealth. J Telemed Telecare.

[17] Johnsen E, Breivik E, Myrvang R, Olsen F (2006). Benefits from telemedicine in Norway. An examination of available documentation.

[18] Uldal SB (1999). A survey of Norwegian telemedicine. J Telemed Telecare.

[19] Knarvik U, Bach B, Lindberg PC, Halvorsen Engeseth K, Skorpen S, Lyngved K (2004). Telemedk@rt 2003 – a survey of telemedicine activities in Norway. http://www.telemed.no/getfile.php/86872.357/Telemedk%40rt2003.pdf.

[20] Norum J, Pedersenwz S, Størmer J, Rumpsfeld M, Stormoww A, Jamissenzz N (2007). Prioritisation of telemedicine services for large scale implementation in Norway. J Telemed Telecare.

[21] Normann T, Breivik E, Skipenes E, Christiansen EK, Knarvik U (2011). Bringing telemedicine into routine service – prerequisites and actions. http://www.telemed.no/getfile.php/1742174.357.vpvfpebvdr/Rapport_Telemedisin+i+rutinedrift_02-2011.pdf.

[22] Jackson DE, McClean SI (2012). Trends in telemedicine assessment indicate neglect of key criteria for predicting success. J Health Organ Manag.

[23] Hanney SR, González-Block MA (2011). Yes, research can inform health policy; but can we bridge the ‘Do-Knowing It's Been Done’ gap?. Health Res Policy Syst.

[24] Sosial-og helsedirektoratet (2011). Innsatsstyrt finansiering 2012. http://www.helsedirektoratet.no/publikasjoner/regelverk-innsatsstyrt-finansiering-2012/Publikasjoner/regelverk-innsatsstyrt-finansiering-2012.pdf.

[25] Helse-og omsorgsdepartementet (2008). LOV 2008-06-20 nr 44: Lov om medisinsk og helsefaglig forskning (helseforskningsloven). http://www.lovdata.no/all/hl-20080620-044.html.

[26] Justis-og beredskapsdepartementet (2000). LOV-2000-04-14-31: Lov om behandling av personopplysninger (personopplysningsloven). http://www.lovdata.no/all/nl-20000414-031.html.

[27] Rogers EM (1983). Diffusion of innovations.

[28] Patterson V, Humphreys J, Henderson M, Crealey G, Patterson V, Humphreys J (2010). Email triage is an effective, efficient and safe way of managing new referrals to a neurologist. Qual Saf Health Care.

[29] Wootton R, Bahaadinbeigy K, Hailey D (2011). Estimating travel reduction associated with the use of telemedicine by patients and healthcare professionals: proposal for quantitative synthesis in a systematic review. BMC Health Serv Res.

[30] Mars M, Scott R (2012). Telemedicine service use: a new metric. J Med Internet Res.

[31] Alkmim MB, Figueira RM, Marcolino MS, Cardoso CS, Pena de Abreu M, Cunha LR (2012). Improving patient access to specialized health care: the Telehealth Network of Minas Gerais, Brazil. Bull World Health Organ.

[32] Blanchet K (2005). The Arizona Telemedicine Program. Telemed J E Health.

[33] Brewer R, Goble G, Guy P (2011). A peach of a telehealth program: Georgia connects rural communities to better healthcare. Perspect Health Inf Manag.

[34] Brown EM (2013). The Ontario Telemedicine Network: a case report. Telemed J E Health.

[35] Kokesh J, Ferguson AS, Patricoski C (2011). The Alaska experience using store-and-forward telemedicine for ENT care in Alaska. Otolaryngol Clin North Am.

[36] Meyers L, Gibbs D, Thacker M, Lafile L (2012). Building a telehealth network through collaboration: the story of the Nebraska Statewide Telehealth Network. Crit Care Nurs Q.

[37] Oliveira TC, Bayer S, Gonçalves L, Barlow J (2014). Telemedicine in alentejo. Telemed J E Health.

[38] Armstrong AW, Wu J, Kovarik CL, Goldyne ME, Oh DH, McKoy KC (2012). State of teledermatology programs in the United States. J Am Acad Dermatol.

[39] Hage E, Roo JP, van Offenbeek MA, Boonstra A (2013). Implementation factors and their effect on e-Health service adoption in rural communities: a systematic literature review. BMC Health Serv Res.

[40] Hendy J, Chrysanthaki T, Barlow J, Knapp M, Rogers A, Sanders C (2012). An organisational analysis of the implementation of telecare and telehealth: the whole systems demonstrator. BMC Health Serv Res.

[41] Sanders C, Rogers A, Bowen R, Bower P, Hirani S, Cartwright M (2012). Exploring barriers to participation and adoption of telehealth and telecare within the Whole System Demonstrator trial: a qualitative study. BMC Health Serv Res.

[42] Smith AC, Armfield NR, Croll J, Gray LC (2012). A review of Medicare expenditure in Australia for psychiatric consultations delivered in person and via videoconference. J Telemed Telecare.

[43] Nutley T, Reynolds HW (2013). Improving the use of health data for health system strengthening. Glob Health Action.

[44] Hahn D, Wanjala P, Marx M (2013). Where is information quality lost at clinical level? A mixed-method study on information systems and data quality in three urban Kenyan ANC clinics. Glob Health Action.

